# Pursuit of Knowledge under Difficulties

**Published:** 1885-04

**Authors:** P. P. Wells


### HOMŒOPATHIC PHYSICIAN.

A MONTHLY JOURNAL OF MEDICAL SCIENCE.

"If our school ever gives up the strict inductive method of Hahnemann, we are lost, and deserve only to be mentioned as a caricature in the history of medicine."—CONSTANTINE HERING.

Vol. V. APRIL, 1885. No. 4.

### PURSUIT OF KNOWLEDGE UNDER DIFFICULTIES.

P. P. WELLS, M. D.

" *Who is our guide, and where shall we find him?*"—HOM. PHYS., Vol. V, p. 174.

The embarrassment felt by the writer of the above quotation has been more or less the experience of us all, at some time in our professional career, when before bewildering examples of sickness we have been called on to relieve. Which of the many drugs showing symptoms in their pathogenesis like those of our case shall we give for the cure? How shall we decide? And for an answer we have turned to what this, that, and the other have written, and perhaps, after all, we have been left, like him who wrote the above, to the unsatisfactory inquiry—" *Who* is our guide, and *where* shall we find him?"

The first answer we have to this query is, if by this "*who*" you mean to ask for the *man* who is to relieve you of your difficulty, *there is no such man*, and therefore he is to be found *nowhere.* In a little different phrase the inquiry may be better expressed, perhaps, and thus: Where and who is the man who will do this, *my* work, for me? Don't ask any more, for he can never be found. This world is so made up, and especially this homœopathic world of ours, that each man in it must *do his own work*, or it is likely to be left undone. If for this he yields to the impulse and incurs the habit of running to his neighbor to do it for him, he may escape a present embarrassment, but he has gained no more, but rather has less, strength with which to encounter the next. This path is only a direct way to personal and professional imbecility.

There is a guide, but it is not found as a man, but only in the form of a law, and is found, if at all, only in the *Organon of Homœopathic Medicine.* The most perfect acquaintance with this law, and the most loyal and constant obedience to it in all clinical duties, is our most perfect emancipation from the embarrassment which seems to have pressed so heavily on our searcher for a "guide." Let him be assured that with proper patience and perseverance he can find this, and find it equal to all his needs. In order to realize this result, he is never to resort to methods outside of law which may tempt by promise of "short and easy" ways to relief and cure, or to any departure from the instructions of this law. This resort to spurious means (palliatives), because apparently their use is to be less a trouble than to find the true specific under the guidance of law, if practiced, is the most perfect hindrance to finding the "guide" this embarrassed one is seeking. Law so disregarded and transgressed, the finding of the "guide" is simply impossible. The transgressor is sure, in the end, to find himself not only "*almost lost*," but utterly so.

The last embarrassment which oppressed this seeker he gives in this manner :

"The patient complained of cold heels, which sweat offensively and profusely in summer. I looked in my guides in vain for the thread. * * * I received my *Medical Advance* * * * and Dr. H. N. Guernsey gave Bar. c., Graph., Kali c., Nit. ac., Sep., Thuja, and Selen., with offensive and profuse sweat of the feet, while Lippe gives Sil. only, and Allen Sil. and Graph."

Now, surely, if one takes repertories as "guides," and stops at them, there is enough to puzzle any man. Repertories are only indices pointing, not necessarily to the specific remedy, but rather to portions of the *Materia Medica* which are to be consulted and studied that this may be found. And then if, under the guidance of law, he has been taught by this not to stop at the facts of cold heels and sweating feet, etc., but with these to gather all the aberrations in the functions of his patient's life from that standard balance we call health, and then to see whether either of these drugs named, or some other, has greatest likeness to this *whole*, he is safe, when he has found this, to accept it as the specific for his case. Anything less than this is only leaving clinical duties partially performed, and this can only often end in disappointment and failure. A practice based on repertories is always weak, uncertain, and unsafe. The practical habit of always referring the repertorial mention of drugs to the *Materia Medica* record for verification should always be cultivated and never be permitted to fall into neglect. It gives strength to duty, and crowns duty with success. Don't stop on the repertory, and don't be discouraged if on going from the repertory to the *Materia Medica* you sometimes fail to find in this last any justification of the mention of the drug for the needs of your present work. This happens oftener than it should, and oftener in large works which have been too hastily prepared, and in this is a chief reason why search for a specific remedy should never stop at them.

But this inquirer has other difficulties, which he expresses thus:

"Now, it seems to me that it is not an eliminated *Materia Medica* that we want, but one containing just this class of symptoms which enables just such men as Guernsey, Lippe, Kent, Bayard, etc., to master their knotty cases. A repertory and *Materia Medica* with this class of information would be an acquisition.

"Take the case of Dr. Kent, in which he cured the spasms of the face. My armamentarium is silent upon such fine discriminations. If any one in the profession can put those who are novices in possession of just this class of information, it will be a star in their crown. The work accepted by Drs. Dake and Hughes may be useful to some, but I cannot see how it would be useful in such cases as those referred to."

This difficulty has come from a mistaken view of the facts in the case. We do not suppose the gentlemen named use different repertories or *Materia Medica* from those in possession of this writer. It is not a difference of books, but a different use, probably, of the same books which enables these prescribers to deal successfully with cases which to this writer are perhaps sometimes "knotty." The information by which they are guided to their successes, and which this writer so earnestly desires, is, no doubt, all in his own possession. The difference is, these gentlemen know where and how to find it. This the writer has not yet learned. He need not be discouraged therefore. These gentlemen had to *learn* the lesson before they *knew* it. This knowledge comes to no one "by nature," except to the Dogberrys, and they do not make the best practitioners of specific medicine. This knowledge only comes as a result of hard work, and much of it. And this, persevered in, will bring it. This work, and not new books, is what is wanted.

"* * * * is a writer I love to read after, and yet he so frequently leaves out the key to his case, etc., * * * * ——, do not forget we need clear-cut work, showing out like Dr. Kent's reports."

"Clear-cut work" is good, and the more of it the better.

But the mistake of this writer is in his desire that some one else shall do this work for him. The successful prescribers whom he names have each done this for himself, and hence their successes. No man could have done it for them, and therefore they stand with us to-day with their present acknowledged ability as specific prescribers. If it could have been done for them by another, the result would have been to leave them afterward the same needy weaklings they were before they were helped, no stronger for the next difficulty by reason of strength acquired by using their own powers in overcoming that just passed. The conclusion of this is a principle of universal application and importance, viz.: No one can do another's man's work for him and not at the same time do him a fundamental injury.

As to the Doctor who "leaves out the key to his case," we think we know something of his motives and plans when he writes, and it has been no part of these to do, in carrying these out, this other man's work. It has been more his object in what he has written of practical Homœopathy to show what *can be done* under its guidance in the first place; and, in the second, to show *how it is to be done* by the other man himself. In the case referred to by this inquirer for a "guide," the object was to demonstrate an important principle in pathology—the fact of the sycotic miasm, and the necessary recognition of this and of the means adapted to its removal before the hitherto partial successes in the treatment of this case could be followed by a complete cure, and also to show how this sycosis was introduced into the life of this child.

These were the objects of this paper, and on reading it again we do not perceive anything is wanted or "left out" which could have made the showing more complete. If the inquirer had found his "guide," he would very likely have shown the "key to the case" was not the drug which cured it, but the *oozing wart* which disclosed a knowledge of the relationship of the drug to the cure, and also the sycotic nature of the case cured. The *key* was not left out, it was only not recognized by our seeker of a "guide." It was no part of his intent to give, in the report of this case, a model to be imitated by others in treating cases they may regard as similar to this. This idea of advantage to any one from reporting cases as models for imitation is wholly misleading and mischievous. It was a consciousness of this which dictated withholding the name of the drug which cured the case so satisfactorily. This was to this inquirer, apparently, "*the key to the case*" he missed. It was a matter of no importance as to the objects of the paper.

We are under obligation to this inquirer for the opportunity he has given us to express our views thus briefly of the relative duties of teacher and pupil. It is no part of the duty of the teacher, either by the pen or from the rostrum, to do the work of the learner for him. He has done his utmost and the best possible when he has shown the neophyte how to do it for himself. To set the pupil to observe and compare facts with his own powers, and show him how this is to be done, and then by the same powers how to select for himself the required curative, under the guidance of these facts and laws—here, in a nutshell, is the whole duty of the true teacher, and herein is all of good the pupil can receive from him. Of course, this is said of the teacher of practical Homœopathy and of the pupil who is seeking a knowledge of its philosophy and the art of its application in practical healing.

